# In vitro tissue-engineered adipose constructs for modeling disease

**DOI:** 10.1186/s42490-019-0027-7

**Published:** 2019-10-29

**Authors:** Connor S. Murphy, Lucy Liaw, Michaela R. Reagan

**Affiliations:** 1 Maine Medical Center Research Institute, Scarborough, ME USA; 2 University of Maine Graduate School of Biomedical Science and Engineering, Orono, ME USA; 3 School of Medicine, Tufts University, Boston, MA USA; 4 Center for Molecular Medicine and Center for Translational Research, 81 Research Drive, Scarborough, ME 04074 USA

**Keywords:** In vitro models, Tissue engineering, Tissue-engineered adipose tissue, Adipocytes, 3D culture, Fat, Cancer, Obesity, Type 2 diabetes

## Abstract

Adipose tissue is a vital tissue in mammals that functions to insulate our bodies, regulate our internal thermostat, protect our organs, store energy (and burn energy, in the case of beige and brown fat), and provide endocrine signals to other organs in the body. Tissue engineering of adipose and other soft tissues may prove essential for people who have lost this tissue from trauma or disease. In this review, we discuss the applications of tissue-engineered adipose tissue specifically for disease modeling applications. We provide a basic background to adipose depots and describe three-dimensional (3D) in vitro adipose models for obesity, diabetes, and cancer research applications. The approaches to engineering 3D adipose models are diverse in terms of scaffold type (hydrogel-based, silk-based and scaffold-free), species of origin (*H. sapiens* and *M. musculus*) and cell types used, which allows researchers to choose a model that best fits their application, whether it is optimization of adipocyte differentiation or studying the interaction of adipocytes and other cell types like endothelial cells. In vitro 3D adipose tissue models support discoveries into the mechanisms of adipose-related diseases and thus support the development of novel anti-cancer or anti-obesity/diabetes therapies.

## Background

The road to US Food and Drug Administration approval for a new pharmaceutical is long and arduous; the process often lasts for over 10 years, costs an average of 2.8 billion dollars, and has only a ~ 10% success rate [1, 2]. This low success rate can be attributed to the fact that drugs often exhibit different effects in clinical trials than in earlier stages of the pipeline (e.g. animal and two-dimensional (2D) cell culture assays). Although 2D cell culture has contributed to the majority of discoveries in modern day cell biology, these cultures do not model the microenvironment cells encounter naturally: a three-dimensional (3D) matrix with abundant cell-to-cell interactions, unique mechanical properties, and an extensive extracellular matrix. Tissue-engineered 3D models have therefore been used in an attempt to more accurately model the in vivo microenvironment. Indeed, various 3D models of cancer, adipose tissue, and many other tissues have been shown to more closely mimic their in vivo counterparts in terms of cellular morphology and global expression profiles [3–5]. This review aims to assess recent advances in 3D modeling for diseases centered on adipose tissue such as: obesity, type 2 diabetes, and cancer adjacent to adipocytes. Although 3D adipose models are also used for soft tissue reconstruction after physical trauma or tumor resections, these topics are outside the scope of disease-based models covered in this review, but are thoroughly reviewed elsewhere [6–8].

## Main text

### Distinct adipose tissues and their functions

Much like the waistlines of the world’s population, the literature regarding the distinct types of adipose tissue has been expanding over the years. The growth in literature is partially due to the discovery of 5 distinct adipose tissue types: white adipose tissue (WAT), brown adipose tissue (BAT), beige/brite adipose tissue, bone marrow adipose tissue (BMAT), and perivascular adipose tissue (PVAT). Given the rich literature on the subject and the limited scope of this review, we will only highlight a few major features of each depot here. A more detailed account of each depot can be found in these comprehensive reviews [9–13].

### White adipose tissue (WAT)

Adipocytes are the primary cellular component of adipose tissue, but adipose tissue is also composed of nervous and connective tissue and vasculature. Adipocytes found in WAT possess the morphology commonly associated with a fat cell; a cell with one large lipid droplet (LD), which occupies the majority of cellular space, forcing the nucleus and cytoplasm to the periphery of the cell. The two main WAT depots, visceral (VAT) and subcutaneous (SAT), are functionally different. VAT is essential for the protection of inner organs and a major contributor to obesity [14, 15]. Conversely, SAT is primarily responsible for insulation and has been associated with improving insulin sensitivity [16]. Although VAT and SAT appear to have opposing functions, they both regulate energy homeostasis. For energy homeostasis, exogenous energy sources such as glucose are stored as glycogen and subsequently converted into triacylglycerols (TGs), the major component of the energy storing organelle, the lipid droplet, in adipocytes [17]. As the cellular need for energy increases, lipases release TGs causing lipid droplets to degrade, in a process known as lipolysis. TGs are subsequently catabolized into glycerol and fatty acids, which eventually produce adenosine triphosphate (ATP) through glycolysis or β-oxidation, respectively [18].

The connection between adipocytes and insulin make adipocytes one of the most significant cell types to regulate systemic insulin levels [19]. Insulin bears much of the responsibility for promoting the cellular uptake of glucose through upregulating the glucose transporter, GLUT4 [20]. To promote energy storage, insulin also inhibits lipolysis through the inhibition of protein kinase A (PKA) [21], and it is thus integral for adipocyte maintenance and function [22]. Adipose vascularization and innervation also reflect the crosstalk that occurs between adipose and distant tissues. For decades, adipocytes were assumed to simply function as cells that store and release energy, and provide mechanical and thermal insulation. However, the complexity of adipocytes was revealed when substances specifically secreted by adipocytes, known as adipokines, were identified in the 1980’s and 90’s [23–25].

The adipokine leptin was found to regulate feeding, fatty acid utilization, and energy balance by serving as a feedback mechanism between adipose and other tissues throughout the body [26–28]. Leptin activates 5′ adenosine monophosphate-activated protein kinase (AMPK), leading to an increase in fatty acid oxidation and inhibition of the rate-limiting step to lipogenesis, acetyl-CoA carboxylase (ACC) action [29]. Leptin also serves to protect against lipotoxicity by shuttling fatty acids away from non-adipose tissue [30]. Another key adipokine, adiponectin (ADIPOQ), is expressed exclusively by mature adipocytes, supports insulin sensitivity, and has a protective effect against cardiac hypertrophy and atherosclerosis [31–33]. Indeed, adiponectin receptors 1 and 2 (ADIPOR1 and ADIPOR2) have anti-diabetic effects [34]. The broad effects of ADIPOQ on metabolism and cardiac health can be attributed to its formation of trimers, hexamers, and even higher molecular weight forms of the protein [35]. Interestingly, each ADIPOQ complex can act on different pathways; for example, while the trimeric form activates AMPK, the higher molecular weight form activates nuclear factor kappa-light-chain-enhancer of activated B cells (NF-κB) [36, 37]. In addition to leptin and adiponectin providing evidence for adipose tissue as an endocrine organ, the relationship between WAT and the reproductive system highlights this relationship as well.

WAT and the reproductive system undergo extensive bidirectional communication mainly through testosterone and estrogen and have profound effects on one another. Dihydrotesterosterone (DHT) can induce lipolysis and inhibit mesenchymal stem cell (MSC) differentiation into adipocytes [38, 39]. Additionally, knocking out the estrogen receptor can lead to white adipocyte hyperplasia and hypertrophy, while knocking out both the estrogen and androgen receptors results in a global increase in adiposity [40, 41]. WAT influences sex hormone levels as well. White adipocytes can inactivate DHT through the action of alpha-keto reductase family member 1 C2 [42–44]. Also, the loss of function of aromatase, the enzyme that converts testosterone to estradiol, can lead to increased abdominal adiposity and insulin resistance [45]. Adipokines like leptin and adiponectin have been shown to influence reproduction as well [46, 47]. The reproductive system and WAT communicate through the regulation and processing of two major hormones, testosterone and estrogen, and have major effects on human health.

### Brown adipose tissue (BAT) and beige adipose tissue

Brown adipose tissue (BAT), an adipose depot unique to mammals, is found at distinct locations: the major depots of BAT in adult mice and rats can be found in the scapulae and thoracic regions, where they serve as the major source of non-shivering thermogenesis [48]. Similarly, adult human BAT is primarily located in the cervical-supraclavicular depot and is identified by the uptake of ^18^F-fluorodeoxyglucose via positron-emission tomography and computed tomography (PET-CT) due to the propensity of BAT to consume more glucose than other healthy tissues [49, 50]. Unlike WAT, BAT is multilocular and takes advantage of the mitochondrial membrane protein, uncoupling protein 1 (UCP-1), to produce heat instead of adenosine triphosphate (ATP) during the process of fatty acid oxidation [51, 52]. UCP-1 functions by increasing membrane permeability of the mitochondrial membrane in order to disrupt the proton motive force at the heart of ATP synthesis. BAT is characterized by the high expression of PR domain containing 16 (PRDM16), Peroxisome proliferator-activated receptor gamma coactivator 1-alpha (PGC-1α), type 2 deiodinase (Dio2), and UCP-1 [53, 54]. Brown and white fat also differ in their progenitors, with the lineage of BAT being traced to a myogenic precursor which is Pax7^+^/Myf5^+^, while the WAT progenitor is Pax7^−^/Myf5^−^ [53, 55].

Beige fat or inducible brown fat, is a prime example of the dynamism that adipocytes exhibit. Upon prolonged cold exposure or adrenergic signaling, a subset of white adipocytes upregulate UCP-1 and adopt a more BAT-like phenotype, a process termed “browning” [11, 56]. Browning initiates a switch from a unilocular white adipocyte to a beige adipocyte that is multilocular and thermogenic, and has an increased number of mitochondria. Both browning and BAT may be protective from obesity based on observations that browning in obese strains of mice is decreased compared to strains resistant to obesity, while the thermogenic capacity of BAT remains the same [57, 58]. Additional evidence for the protective effects of beige and brown fat suggests they play a major role in decreasing circulating TG and glucose levels [59]. Deeper investigations into brown and beige fat biology will likely help combat metabolic syndromes like obesity and type 2 diabetes mellitus (T2DM) that are reaching epidemic levels in many countries.

### Bone marrow adipose tissue (BMAT)

Peering into the bone marrow within the long bones of an adult human or mouse, one would observe that 50–70% of the bone marrow has distinct yellow hue [60]. This hue is due to the presence of bone marrow adipose tissue (BMAT). BMAT is induced by common medical practices such as the administration thiazolidinediones (such as the anti-diabetic drug, rosiglitazone), radiation, and chemotherapy [61–64]. Studies suggest that BMAT may have a complicated role in global metabolism since BMAT is increased both in obesity and paradoxically, in patients suffering from anorexia nervosa or starvation [65, 66]. BMAT appears to have a distinct lineage from other adipose depots; bone marrow MSCs that are CD45^−^/CD31^−^/PdgfRa^+^/Sca1^+^ differentiate into adipocytes [67]. Additionally, BM adipocytes originate from osterix^+^ cells while other adipocytes do not [68]. The role of BMAT with respect to global metabolism and the effects of BMAT on the local bone marrow microenvironment remain important questions to address in the field.

### Perivascular adipose tissue (PVAT)

**Fig. 1:**
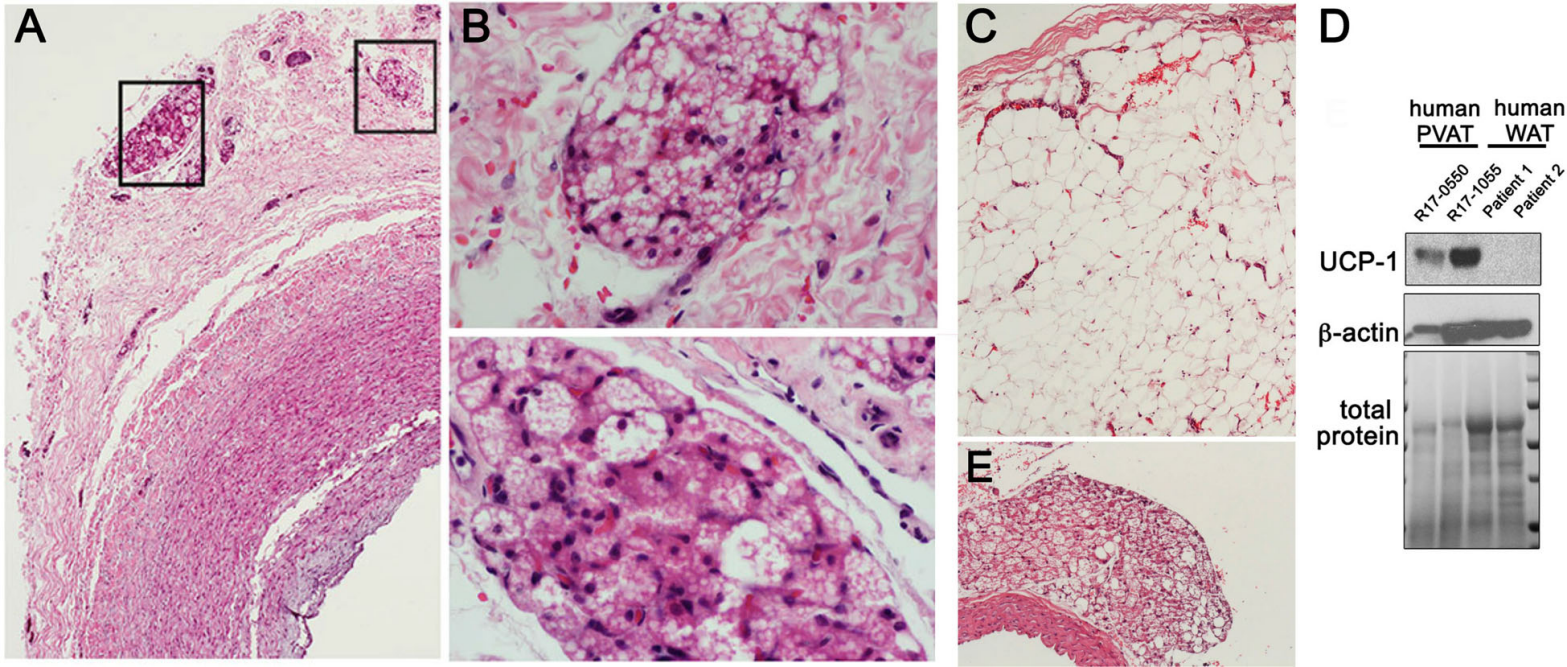
Human aortic PVAT has features of thermogenic adipose tissue. **a** Shown is a carotid artery with surrounding adventitia and PVAT from a 1-month old human donor. **b** Note the pockets of brown-like adipose tissue (boxed), that are morphologically indistinguishable from brown adipose tissue. **c** Human PVAT surrounding aorta was collected from an adult during open-heart surgery, and morphologically resembles WAT. However, compared to subcutaneous human WAT, human aortic PVAT, even from patients with cardiovascular disease, express the thermogenic adipocyte marker UCP-1. **d** Western immunoblot of human PVAT and subcutaneous WAT for the indicated proteins. R17–0550 and R17–1055 represent samples from two different patients. Reprinted by permission from RightsLink: Springer Nature, *Cardiovascular Drugs and Therapy*, Boucher et al. 2018. **e** Mouse PVAT from the thoracic aorta is shown for comparison, and has a brown fat-like thermogenic phenotype and protein profile

Perivascular adipose tissue, the fat depot adjacent to the adventitia of most arteries, is an integral signaling component of the vascular microenvironment. Expansion of PVAT is associated with obesity and cardiovascular disease in humans, with pathological changes described in patients with localized vasospasm, abdominal aortic aneurysm, and coronary artery disease [69–72]. In addition to its basal ability to store and release fatty acids, PVAT alters: vascular tone, smooth muscle cell proliferation and migration, inflammatory programs, and oxidative stress pathways. PVAT exerts its influence on the surrounding tissues through the secretion of adipokines and cytokines such as leptin, adiponectin, TNF-α, and IL-6 [73–76]. Although healthy PVAT is considered vasoprotective, obesity and hyperlipidemia induce changes that can promote vascular disease progression. In a genetic mouse model of atherosclerosis, the PVAT from a apolipoprotein E-null (ApoE^−/−^) mouse promoted atherosclerotic plaques in a region where it does not usually form in ApoE^−/−^ mice [77, 78]. The phenotype of mouse and human PVAT depends on its location. While PVAT near the carotid artery adopts a BAT-like morphology (Fig. 1a and b) it more closely resembles WAT in the mesenteric arteries (Fig. 1c). Indeed, the thermogenic properties and BAT-like expression pattern of thoracic aorta-associated PVAT proved to attenuate atherosclerosis [79], suggesting PVAT as an important source of paracrine regulation in vascular disease. There is some question about how well mouse models of PVAT expansion and pathology can mimic these processes in humans, since aortic PVAT derived from adult humans is morphologically more similar to WAT than BAT. However, it is clear that human aortic PVAT, even from patients with cardiovascular disease, express thermogenic markers including UCP-1, which is absent in human WAT (Fig. 1d). Within the thoracic aorta, PVAT adopts a more BAT-like phenotype (Fig. 1e) [79, 80]. Studies of human PVAT have shown that PVAT-derived adipocyte progenitor cell differentiation is dependent on Rab27a, a GTPase important for secretory vesicle trafficking [81]. One could imagine that the bi-directional communication between PVAT and the blood vessel relies on Rab27a-dependent trafficking and secretion of signaling molecules. These interesting results highlight that the full potential of targeting PVAT with respect to cardiovascular disease and obesity has yet to be realized and requires further study.

As researchers delved deeper into adipocyte biology, they have come to appreciate that adipocytes are highly sensitive to metabolites, cytokines, and hormones, and can also regulate processes like angiogenesis, inflammation, immunity, reproduction, and cardiac homeostasis [25, 82–89]. Given that adipose tissue is intimately intertwined in a diverse number of biological processes, it is imperative to understand the intricacies of this amazing tissue.

## Adipose-associated disease models

### Background on 3D models

Tissue engineers must consider a number of key characteristics that are conducive to constructing 3D tissues in vitro. Pore size is a parameter that positively correlates with nutrient availability and modulates cell proliferation, migration, and matrix deposition. Researchers must also consider the ideal mechanical stiffness for their cell type in question and how that compares to the in vivo environment they are attempting to model. Additionally, the material’s degradation properties should be predictable and be able to be manipulated in order to assess the kinetics of culturing cells on that material. Understanding the degradation rate is an important experimental parameter because it can factor into how long cells are able to be cultured. Importantly, the material in question should be biocompatible and induce a cellular response that best reflects that found in vivo [90–92]. In order to facilitate the study of disease states, cell viability and growth must be able to be maintained for long-term culture. Together, these and other parameters of 3D systems must be considered and effectively designed for researchers to successfully create 3D-based disease models.

### Obesity and type II diabetes mellitus

Obesity is a worldwide health concern that affects roughly a third of the population and costs an estimated $209.7 billion dollars per year in the United States alone [93, 94]. Obesity is defined as having a body mass index (BMI) of > 30 kg/m^2^, is characterized by excess accumulation of adipose tissue, and is a major risk factor for various health issues including T2DM, cardiovascular disease, and some cancers [95–97]. Obesity has been difficult to study because the regulation of adipocytes is influenced by environmental, genetic, and epigenetic factors, but the development of tissue-engineered models could aid in providing insight into this complicated disease [98–100].

**Fig. 2:**
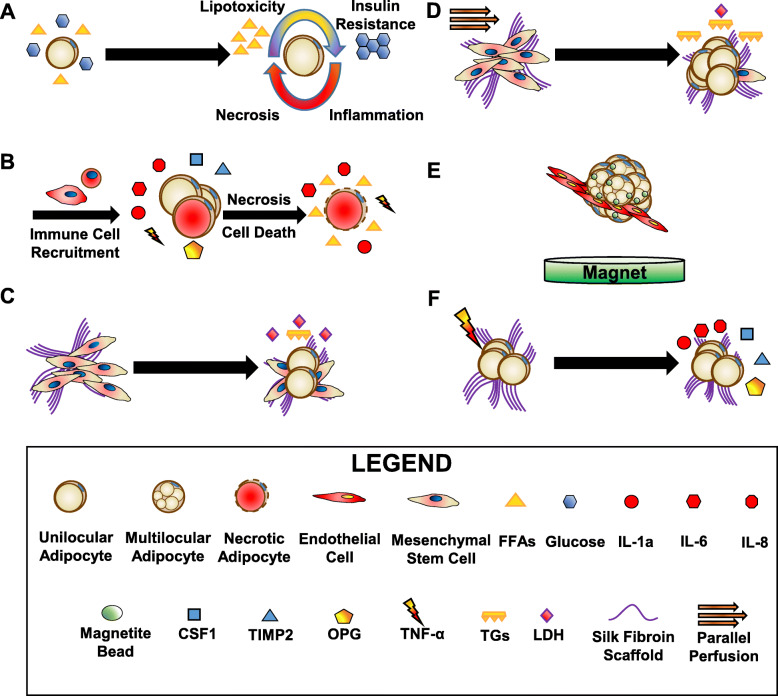
Overview of the effects of obesity on adipocytes and 3D tissue-engineered adipose models of obesity. **a** During obesity, excess calories (whether from free fatty acids (FFAs) or glucose) cause adipocytes to become hypertrophic. The increase in FFAs causes activation of oxidative and endoplasmic reticulum (ER) stress and subsequent secretion of cytokines and adipokines. Oxidative and ER stress cause insulin resistance by negatively regulating insulin signaling. As inflammation and adipocyte size increase, oxygen is unable to penetrate the adipose tissue causing hypoxia, necrosis and eventually cell death. **b** Hypertrophic adipocytes secrete chemokines (C-C motif chemokine ligand 2 (CCL2), CCL8, CCL5, colony stimulating factor 1 (CSF1)) and cytokines that attract immune cells, mainly macrophages. Adipose tissue macrophages can secrete anti-inflammatory factors (interleukin-10 (IL-10) and IL-1) and pro-inflammatory factors (tumor necrosis factor α, (TNF-α), IL-6 and IL-1β). Inflammation can cause necrosis and cell death, further releasing pro-inflammatory molecules like cytokines and excess lipids, perpetuating the cycle of chronic low-grade inflammation. **c** and **d** Bellas et al. 2013 and Abbott et al. 2015 both demonstrated the benefits of perfusion (**d**, yellow-orange arrows) compared to static culture (**c**) for human mesenchymal stem cell (hMSC)-derived adipocytes differentiated on silk fibroin scaffolds (e.g. increased differentiation, triacylglycerols (TGs), and viable culture time, and decreased the damage-associated protein, lactate dehydrogenase (LDH)). **e** Daquinag et al. 2013 co-cultured 3 T3-L1 preadipocytes with the endothelial cell line bEND.3 embedded with magnetite nanoparticles. **f** Abbott et al. 2016 obtained adipocyte-derived stem cells from lipoaspirates and seeded and differentiated these cells on silk scaffolds. These cultures secreted factors found in both obesity (IL1-α, osteoprotegerin (OPG), and tissue inhibitor of metalloproteinases 2 (TIMP2)) and type 2 diabetes mellitus (IL-6 and IL-8)

In an effort to increase the capacity of energy storage during caloric excess, adipocytes increase in size, a phenomenon called hypertrophy. Although increases and decreases in adipocyte size based on the nutrition state of the organism is normal, excessive and prolonged adipocyte hypertrophy is often a sign for adipocyte dysfunction and leads to system-wide changes due to an altered adipocyte secretome. Although hypertrophic adipocytes have an increased capacity to store lipids, it is insufficient to contend with the extracellular excess of free fatty acids (FFAs). The excess of FFAs induces local lipotoxicity from increases in oxidative, and endoplasmic reticulum (ER) stress and can have system-wide effects as well (Fig. 2a) [101, 102]. The increase in size of adipocytes prevents oxygen from diffusing across the adipose tissue, resulting in a hypoxic microenvironment [103–105]. Together, adipocyte hypertrophy, hypoxia, oxidative and ER stress contribute to the increase in the secretion of adipokines and cytokines as well as the differentiation of new adipocytes [106, 107]. An increase in local pro-inflammatory cytokines resulting from adipocyte secretion, necrosis, and lysis leads to immune cell recruitment and chronic inflammation, perpetuating adipocyte dysfunction (Fig. 2b) [108, 109]. When compared to healthy patients, obese individuals also have increased leptin, fasting glucose, TGs, inflammatory markers, insulin concentrations, and decreased high-density lipoproteins (HDLs) in their serum [110]. Together, these factors can lead to additional morbidities including T2DM and cardiovascular disease. Interestingly, there is a subpopulation of obese patients that have an increase in fat mass but lack the risk of metabolic dysfunctions and cardiovascular disease, known as metabolically healthy obese patients [110]. For the purposes of this review, obesity will refer to the metabolically dysfunctional population.

Impaired insulin signaling is at the heart of why type 2 diabetes patients fail to regulate their blood sugar and is intimately linked with obesity [95]. Insulin signaling activates two major pathways through phosphorylation of insulin receptor substrates (IRSs) on tyrosine residues: (1) the phosphatidylinositol 3-kinase (PI_3_K)-AKT and (2) mitogen activated protein kinase (MAPK) pathways [111]. While the PI_3_K-AKT pathway regulates glucose uptake and suppresses gluconeogenesis, the MAPK pathway interacts with PI_3_K-AKT to regulate cell growth and differentiation. Insulin signaling is negatively regulated by phosphorylation of serine residues on IRS1 by kinases such as I kappa B kinase beta (IKK-β) and C-jun-N-terminal kinase (JNK1) [111]. Interestingly, ER stressors from FFA signal through IRE1 to activate both IKK and JNK pathways, contributing to insulin resistance. Additionally, blood glucose remains higher in obese individuals due to *GLUT4* expression in adipocytes being downregulated by the induction of ER stress [112]. The negative effects of inflammation are further perpetuated by the recruitment of adipose tissue macrophages (ATMs).

Activated stress pathways found in obesity drive the expression of chemokines like C-C motif chemokine ligand 2 (CCL2), CCL8, and CCL5, and chemokine receptors (e.g. CCR2 and CCR5), which help recruit macrophages to the dysfunctional tissue [108, 113]. ATMs form crown-like structures around the stressed adipocytes in order to clear debris and excess FFAs [108]. The recruited ATMs secrete both anti-inflammatory (interleukin-10 (IL-10) and IL-1) and pro-inflammatory (tumor necrosis factor-α (TNF-α), IL-6, and IL-1β) cytokines, suggesting they can serve both a protective and harmful role in obesity [114]. However, the inflammation caused by ATMs causes further adipocyte dysfunction and impaired insulin signaling, leading to the manifestation of chronic low-grade inflammation. Despite the complexity of obesity and the early stages of tissue-engineered adipose disease models, researchers have made important strides to move the field forward.

### Current 3D adipose disease models

An ideal tissue engineered 3D adipose tissue model would be comprised of each component found in vivo: adipocytes, connective tissue, vasculature, and nerves. Given that determining the ideal conditions for one cell type is technically challenging, the addition of more cell types compounds the complexity of the model. While groups have engineered 3D nervous tissue models, the interaction between adipocytes and nervous tissue in 3D system has been understudied [115–118]. However, the following 3D adipose models could be adapted in the future to support the growth of both cell types. Groups have primarily focused efforts on engineering systems of adipocytes on scaffolds that mimic in vivo connective tissue or scaffold-free models in which the adipocytes secrete their own extracellular matrix with and without endothelial cells. Therefore, the following section will discuss current avascular and vascular 3D adipose disease models in cells derived from both mouse and humans. Please refer to Table 1 for details on each paper cited below regarding adipose-related disease models.

**Table 1:** 3D Adipose Models of Obesity, Type II Diabetes Mellitus and Cancer

Publication	Organism	Source of Cells and Disease Context	Scaffold	Advantages over 2D / Major Findings
Emont et al. 2015 [119]	*M. musculus*	Inguinal Fat Depot, Perigonadal Fat Depot, SVF Obesity	Collagen Hydrogel	↑Expression: *Adipoq* *Fabp4* *Pparg* Distinct expression patterns between inguinal and perigonadal fat depots
Davidenko et al. 2010 [120]	*M. Musculus*	3 T3-L1 s (pre-adipocytes) Obesity	Freeze Dried Collagen-Hyaluronic Acid Crosslinked with EDC and NHS	↑ 3 T3-L1 Proliferation ↑ *adipsin* ↑ Compressive Strength and Stiffness
Turner et al. 2014 [121]	*M. Musculus*	3 T3-L1 s Obesity	Copolymer of elastin-like polypeptide (ELP) and polyethyleneimine (PEI) to promote spheroid formation	↑ Intracellular TG Content ↑ CD36 ↑ CD40 ↑ Pparg ↑ Adipoq When Exposed to TNFα: ↑ Glycerol Secretion ↑ FFA Concentration in Media
Vidyasekar et al. 2016 [122]	*M. Musculus*	BM-MSCs	PLA microbeads polymerized with gelatin, alginate, dextran and pectin	↑ Lipid Content (Nile Red) ↑ Expression: *Pparg* *Fabp4* Cebp*α* Perfusion Increased above parameters
Kuss et al. 2018 [123]	*H. sapiens*	hTERT immortalized isolates of BAT from a female patient Obesity/T2DM	Photocrosslinkable Hyaluronic Acid and Gelatin	Tunable Mechanical Properties ↑ Expression: *UCP1* *PGC1A* *ZIC1* ↓ Expression: *HDAC1* ↑ Max and Basal Respiration ↓ IL-6 Improved Glucose Uptake +/− Insulin
Hsiao et al. 2016 [124]	*H. sapiens*	ASCs Obesity	collagen and fibrinogen encapsulated within an alginate cylindrical shell	Unilocular Adipocytes ↑ Expression: ADIPOQ
Mauney et al. 2007 [125]	*H. sapiens*	ASCs hMSCs Obesity	Silk	↑ Expression: *FABP4* *PPARγ* *GLUT4* *ADIPSIN* *ACS* ↑ Lipid Content (Oil Red O) Implantable within Mice
Bellas et al. 2013 [126]	*H. sapiens*	ASCs Obesity	Silk	Built on system in Mauney et al. Dynamic Spinning Increased Culture Time to 6 months
Abbott et al. 2015 [127]	*H. sapiens*	ASCs derived from inguinal adipose tissue Obesity	Silk in a PDMS microfluidics device	3D + Perfusion: ↑ TGs ↑ Glycerol Secretion ↓ LDH
Aubin et al. 2015 [128]	*H.* *sapiens*	ASCs Microvascular endothelial cells Obesity	Serum and Ascorbic Acid Stimulated Self-assembled Cell Sheets	Long-term culture Expressed similar to AT explant: Leptin ANGPT1 PAI-1 VEGF HGF Mature endothelial structures
Aubin et al. 2015 [129]	*H.* *sapiens*	ASCs Obesity	Serum and Ascorbic Acid Stimulated Self-assembled Cell Sheets	Exposure to TNF-α: ↑ MCP-1 ↓ *SLC2A4* ↓ *FASN* ↑ NF-κB-related transcripts Dynamic culture increased lipid content
Qi et al. 2018 [130]	*H. sapiens*	hTERT immortalized isolates of WAT from a female patient and HUVECs Obesity	methyacrylated HA, gelatin and PEG-4A-based hydrogel	↑ Expression: *PPARG* *LPL* *CEBPB* Supports co-culture with endothelial cells
Daquinag et al. 2013 [131]	*M. Musculus*	ASCs from the SVF 3 T3-L1 s bEND.3 Endothelial Cells	Magnetically Levitated Cells	Ability to form spheroids Decorin Deposition
Choi et al. 2010 [132]	*H. sapiens*	ASCs HUVECs Obesity/ T2DM	Silk	In co-culture w/ HUVECs and in high insulin: ↓ Intracellular TG Content
Abbott et al. 2016 [133]	*H.* *sapiens*	Liquified Lipoaspirates: Adipocytes, ASCs, fibroblasts, smooth muscle pericytes and endothelial cells Obesity	Silk	↑ Glycerol Secretion ↑ TG Secretion When Exposed to TNFα: ↑ IL1-α ↑ OPG ↑ TIMP2 ↑ IL-6 ↑ IL-8 ↑ RANTES
Das et al. 2015 [134]	*H.* *sapiens*	Adipocytes derived from human nasal inferior turbinate tissue-derived mesenchymal stromal cells Obesity	Silk-gelatin crosslinked with tyrosinase (bioink)	↑ *PPARG* ↑ *CEBPα* ↑ *LPL*
Proulx et al. 2016 [135]	*H. sapiens*	ASCs Microvascular endothelial cells Obesity	Serum and Ascorbic Acid Stimulated Self-assembled Cell Sheets	Upon adipocytes stimulated with TNF-α and IL-β: ↓ EC Length ↓ EC Branching
Proulx et al. 2018 [136]	*H.* *sapiens* *M. musculus*	hASCs human microvascular endothelial cells grafted into nude mice Obesity	Serum and Ascorbic Acid Stimulated Self-assembled Cell Sheets	Integrated with host vasculature within 3 days No significant loss of adipocyte volume after 14 days of implantation.
Yang et al. 2015 [137]*	*H.* *sapiens*	ASCs MCF-7 s Cancer	Microfluidics device	Real-time monitoring of tissue progression, Circulation-like dynamic flow of media, Studying the relationship between light penetration and tissue depth in photodynamic therapy
Dunn et al. 2014 [138]*	*H. sapiens*	Decellularized subcutaneous adipose tissue from patients Cancer	Human adipose tissue derived extracellular matrix	Models ECM of Breast Cancer Tumor Microenvironment
Campbell et al. 2014 [139]	*M. Musculus*	KIM-2 3 T3-L1 s Cancer	Collagen/HA	Recapitulated mammary gland involution
Hume et al. 2018 (a) [140]	*H.* *sapiens*	MSCs (isolated from mammoplasty surgery) MDA-MB-231 Cancer	Collagen I	Allowed: 7 days of co-culture of adipocytes and MDA-MB-231 cells Multiphoton Microscopy Determination of adipocyte-dependent contributions to MDA-MB-231 migration
Hume et al. 2018 (b) [141]	*H.* *sapiens*	3 T3-L1 s MMTV-*Wnt1* transgenic mouse (FVB background) TUBO (derived from Balb/c-Her2/neu transgenic mouse) Cancer	Collagen I	Assess tumor fragment migration ↑ imaging capabilities due to optical clearing MMTV-*Wnt1* ROCK inhibitors promoted MMTV-*Wnt1* migration 72 h Adipocyte co-culture was anti-migratory in MMTV-*Wnt1*
Bougaret et al. 2018 [142]	*H.* *sapiens*	ASCs MCF-7 Cancer	Collagen-glycosaminoglycan-chitosan	MCF-7 cells less sensitive to the effects of tamoxifen when co-cultured with adipocytes from obese women May be due to difference in IL-6, leptin and TNFα
Mosaad et al. 2018 [143]	*H.* *sapiens*	Adipocytes derived from BM-MSCs C42Bs Cancer	Scaffold Free PDMS microwell-mesh	↓ of C42B cell migration in co-culture with adipocytes Lack of docetaxel resistance in C42B cells co-cultured with adipocytes
Herroon et al. 2016 [144]	*M. musculus* *H. sapiens*	Mouse Adipocytes and macrophages derived from BM-MSCs PC3s Cancer	Spheroids grown on Collagen IV	Adipocytes increased spheroid after 3 and 5 days of co-culture
Fairfield et al. 2018 [4]	*M. Musculus* *H. sapiens*	Mouse and Human adipocytes derived from BM-MSCs 5TGM1 (Mouse Myeloma) MM1S (Human Myeloma) OPM2 (Human Myeloma) Cancer	Silk	In Adipocytes, Upregulated transcripts associated with: Proliferation Differentiation Growth Factor Response ECM Secretion Pattern Specification In Adipocytes, Downregulated Transcripts associated with: TLR-signaling COX/COX2 Pathways Lipoproteins

References used in the publication that pertain to 3D adipose models of obesity, type II diabetes mellitus and cancer are presented here with details regarding: organism(s) used in the study, the specific cell types and disease context, the scaffold type and major findings. Studies highlighted with an asterisk (*) do not use differentiated adipocytes. Abbreviations: 1-Ethyl-3-[3-dimethylaminopropyl] carbodiimide hydrochloride (EDC), 4arm polyethylene glycol (PEG-4A), adiponectin (ADIPOQ), adipose-derived stem cells (ASCs), Angiopoietin-1 (ANGPT1), bone marrow-derived mesenchymal stem cells (BM-MSCs), Brown adipose tissue (BAT), CCAAT enhancer binding protein alpha or beta (CEBPα or β), cyclooxygenase (COX), elastin-like polypeptide (ELP), extra cellular matrix (ECM), fatty acid binding protein 4 (FABP4), hepatocyte growth factor (HGF), high-density lipoproteins (HDLs), hyaluronic acid (HA), interleukin 1α (IL-1α), interleukin 1β (IL-1β), interleukin 6 (IL-6), interleukin 8 (IL-8), interleukin 10 (IL-10), lactate dehydrogenase (LDH), lipoprotein lipase (LPL), monocyte chemoattractant protein-1 (MCP-1), mouse mammary tumor virus (MMTV), N-hydroxysuccinimide (NHS), nuclear factor kappa-light-chain-enhancer of activated B cells (NF-κB), Osteoprotegerin (OPG), peroxisome proliferator-activated receptor gamma (PPAR-γ / Pparg), peroxisome proliferator-activated receptor gamma coactivator 1-alpha (PGC-1A), plasminogen activator inhibitor-1 (PAI-1), polydimethylsiloxane (PDMS), poly-lactic acid (PLA), regulated-on-activation-normal-T-cell-expressed-and-secreted (RANTES), rho associated coiled-coil containing protein kinase (ROCK), stromal vascular fraction (SVF), three-dimensional (3D), toll-like receptors (TLR), Triacylglycerols (TGs), tumor necrosis factor alpha (TNF-α), two-dimensional (2D), type 2 diabetes mellitus (T2DM), uncoupling protein 1 (UCP1), vascular endothelial growth factor (VEGF), white adipose tissue (WAT)

### Mouse avascular 3D adipose disease models

The majority of mouse avascular 3D adipose models use novel substrates or a combination of existing ones to assess the physical properties which best support adipocyte differentiation and function. Motivated to develop a 3D method to compare subcutaneous and visceral fat depots, due to the inefficient differentiation of stem cells isolated from visceral fat, Emont et al. adopted a 3D collagen-based cell culture system [119]. Cell suspensions from mouse inguinal, perigonadal, and SVF fat depots were homogenized and mixed with collagen or plated in collagen-coated 2D wells and allowed to differentiate for 6–7 days to determine if the 3D cultures could enhance the differentiation of visceral fat precursors. Emont et al. showed that 3D culture increased expression of genes associated with differentiated adipocytes like adiponectin (*Adipoq*), fatty acid binding protein 4 (*Fabp4*), and peroxisome proliferator-activated receptor gamma (*Pparg)*. Additionally, in their 3D collagen hydrogel, visceral and subcutaneous adipose maintained distinct expression patterns in terms of thermogenesis, cytokine secretion in response to lipopolysaccharide and thermogenic and lipolysis-dependent adrenergic stimulation. Although not studying obesity directly, this 3D collagen-based method could be utilized to model obesity, T2DM, and other adipose-related diseases.

Davidenko et al. characterized the physical properties of a collagen-hyaluronic acid (HA)-based 3D scaffold. Collagen and HA mixtures were made with 7.5 and 15% HA, freeze-dried, and subsequently crosslinked with 1-Ethyl-3-[3-dimethylaminopropyl] carbodiimide hydrochloride (EDC) and N-hydroxysuccinimide (NHS), which facilitates the attachment of glycosaminoglycans (GAGs) to collagen and provides structural support to the scaffold [120]. When compared to collagen scaffolds alone, they found that the pore size ranged from 100 to 220 μm, had a similar degradation time, and had increased compressive strength and stiffness. The collagen-HA scaffolds also increased the proliferation of mouse adipocyte precursors (3 T3-L1 s) and increased expression of the adipokine, *complement factor D* (*adipsin*), although *Pparg* expression was not significantly changed. The determination of the total number of cells, lipid content, insulin signaling, metabolism, and thermogenic capacity of adipose tissue would help solidify this approach as a viable disease model.

In an effort to assess the inflammatory capacity of adipose tissue in a 3D environment, Turner et al. used a copolymer of elastin-like polypeptide (ELP) and polyethyleneimine (PEI) to promote adipocyte spheroid formation [121]. Turner and colleagues showed that differentiated mouse 3T3L-1 spheroids had increased intracellular TG and increased protein expression of the fatty acid uptake regulator CD36, CD40, Pparg, and Adipoq when compared to 2D cell culture. Upon stimulation with TNF-α, there was an increase in both glycerol secretion and concentration of FFAs, indicative of lipolysis. Follow-up proteomics and confirmation of lipolysis would help confirm the ability of researchers to model obesity-related inflammation. While both Turner et al. and Emont et al. analyzed the inflammatory capacities of adipose tissue in their respective 3D models, researchers must be aware that the inflammatory signaling (particularly TNF and beta-3 adrenergic receptor signaling) differ between mice and humans [119, 121, 145–147]. Studies centered on the inflammatory response of current human models are reviewed in the proceeding sections.

Vidyasekar et al. used poly-lactic acid (PLA) microbeads polymerized with gelatin, alginate, dextran, and pectin as a scaffold for mouse bone marrow-derived MSCs (BM-MSCs) to test whether functional pre-adipocytes could differentiate on this substrate [122]. Indeed, the microbeads supported adipogenesis as shown by an increase in Nile Red staining and the upregulation of *Pparg*, *Fabp4,* and *Cebpα*. These parameters were further enhanced by culturing the microbead-associated cells in a rotating container, further supporting the benefits of mechanical forces and perfusion to recapitulate the in vivo environment.

### Human avascular 3D adipose disease models

While the majority of 3D adipose tissue models primarily focus on WAT, Kuss et al. aimed to engineer a photocrosslinkable HA and gelatin-based 3D BAT model to explore the role of brown adipose depots in obesity and T2DM [123]. The authors used bioprinted photocrosslinkable methacrylate to alter both the porosity and stiffness of their scaffolds based on varying the spacing parameters during printing and exposure to ultraviolet light, respectively. The authors showed that immortalized brown adipocytes cultured with angiogenic factors such as vascular endothelial growth factor (VEGF) and fibroblast growth factor 2 (FGF2) before differentiation showed an enhanced brown adipose phenotype by significant upregulation of *Ucp1*, *Pgc1α,* and *Zic1* and a concurrent decrease of a negative regulator of BAT differentiation, *Hdac1*. Immortalized BAT cell lines seeded onto stiff and porous scaffolds showed increased BAT gene expression, basal and maximal respiration, decreased inflammatory markers such as IL-6, and improved glucose uptake in the presence and absence of insulin. Taken together, Kuss et al. demonstrated that the use of methacrylated HA and gelatin supports culturing brown adipocytes and their metabolic activity, and they found that expression patterns can be modulated by modifying the porosity and stiffness of the matrix. This phototunable model allows the possibility of spatial specific differentiation of MSCs into adipocytes, offering the possibility to study the interactions of WAT, BAT, and other cell types on one contiguous scaffold.

On the other hand, Hsiao et al. engineered a flexible corkscrew-shaped model of 3D adipose tissue by seeding human adipose-derived stem cells (ASCs) into collagen and fibrinogen encapsulated within an alginate cylindrical shell [124]. Upon the addition of adipogenic media, clear unilocular adipocytes were observed and an increase in ADIPOQ after 11 days was evident. Additional molecular markers of adipose tissue differentiation and obesity would help determine if this physically flexible model could be used to study adipose-related diseases. The presence of the alginate shell in this model offers the possibility to study the contributions of paracrine and juxtracrine signals of adipocytes on any cell type of interest in vitro and in vivo as an implant.

The Kaplan group was the first to use silk 3D scaffolds as model for tissue-engineered adipose tissue in 2007 [125]. Mauney et al. assessed adipogenesis in four different scaffold biomaterials: silk prepared by aqueous and organic solvent-based processes, collagen, and PLA. Human ASCs (hASCs) and human bone marrow-MSCs (hBM-MSCs) seeded onto silk scaffolds and allowed to differentiate into adipocytes showed expression of genes associated with adipocyte differentiation: *FABP4*, *PPARγ*, *GLUT4,* adipsin, and acyl-CoA synthetase and had accumulated lipids as measured by Oil Red O. In order to demonstrate a clinical application of this approach, silk scaffolds seeded with either ASCs and BM-MSCs that were implanted into mice were able to undergo adipogenesis and were more durable than both the collagen and PLA-based scaffolds. In 2013, Bellas et al. showed that the addition of dynamic spinning turbulence during culture of adipose tissue seeded onto the silk scaffolds described by Mauney et al. could support functional adipocytes for up to 6 months (Fig. 2c,d) [125, 126].

Abbott et al. then developed a parallel perfusion method that utilized a 3D silk scaffold embedded in a polydimethylsiloxane (PDMS) device that had channels that allowed longitudinal flow of liquid across the scaffold (Fig. 2c,d) [127]. Adipocytes cultured on the 3D scaffold under perfusion had increased TGs and glycerol secretion and had a remarkable reduction in the damage associated protein, lactate dehydrogenase (LDH), after 42 days of culture compared to 2D cell culture. Both Bellas et al. and Abbott et al. demonstrate that the addition of dynamic flow to silk scaffolds can functionally improve adipose tissue and increase the longevity of cultures [126, 127]. The long-term culture that silk scaffolds afford offers researchers to gain deeper insight into the progression of adipose-related diseases but is not limited to silk scaffolds.

Interestingly, in a self-assembly cell sheet-based 3D model, Aubin et al. demonstrated healthy culturing of adipocytes to at least 81 days after differentiation [128]. Briefly, human ASCs derived from lipoaspirates were seeded onto paper anchorage discs and induced to deposit their ECM via the addition of serum and ascorbic acid [128, 129]. The cell sheets were stacked on top of one another to the desired tissue thickness. Adipocytes cultured in this model expressed leptin, angiopoietin-1 (ANGPT1), plasminogen activator inhibitor-1 (PAI-1), VEGF, and hepatocyte growth factor (HGF) to similar levels as patient explants [128]. Importantly, Aubin et al. also showed a physiological relevant response to TNF-α stimulation in terms of monocyte chemoattractant protein-1 (MCP-1) expression, downregulation of genes involved in insulin-mediated uptake of glucose and fatty acid synthesis, as well as increased expression in NF-κB-related transcripts [128]. Finally, self-assembled adipose sheets in dynamic culture had increased lipid content as seen by Oil Red O staining after 7, 14, and 21 days after differentiation. Taken together, the model developed by Aubin et al. offers a model to study the inflammatory response of adipocytes and its effect on other cell types over a period of months.

While the addition of dynamic flow may serve the function of increasing nutrient availability like the vasculature, the addition of endothelial cells more accurately models the in vivo microenvironment in terms of cell-to-cell interactions.

### Human vascular 3D adipose disease models

In a complementary study to Kuss et al., the Duan group engineered a methacrylated HA, gelatin, and 4arm polyethylene glycol (PEG-4A)-based hydrogel using cryopolymerization, where controlled freeze-thaw cycles led to enhanced tensile strength and a microporous network. As opposed to traditional hydrogels, the Duan design enables injections of media or reagents multiple times [130, 148]. This cryogel model increased the expression of adipocyte-related genes such as *PPARG*, lipoprotein lipase (*LPL*), and CCAAT Enhancer Binding Protein Beta (*CEBPB*) in a differentiated human immortalized white adipose cell line. The expression of these genes was further enhanced by co-culture of human umbilical vascular endothelial cells (HUVECs). As opposed to the stiff porous scaffold described in Kuss et al., the flexible and porous cryogel method supported the culture of WAT cells with and without other cell types [123, 130].

Daquinag et al. adapted a technique developed by the Pasqualini lab which used a magnetite and gold nanoparticles crosslinked to poly-L lysine to promote endocytosis to magnetically suspend scaffolds and promote 3D spheroid formation (Fig. 2e) [131, 149]. The scaffolds were seeded with ASCs from the stromal vascular fraction (SVF) or the pre-adipocyte mouse cell line, 3 T3-L1, and allowed to differentiate into WAT while being magnetically suspended. The magnetically-levitated scaffolds demonstrated the ability to form a spherical complex with mature adipocytes, CD31^+^ endothelial cells, and the deposition of the ECM protein, decorin. It will be exciting to see how the magnetically-levitated cells are functionally different when compared to both 2D and other 3D adipose models.

Choi et al. aimed to model insulin resistance in a adipocyte and endothelial cell co-culture model on the silk scaffolds described in Mauney et al. [125, 132]. hASCs were plated in 3D mono or co-culture with endothelial cells in the presence of 1 μM or 10 μM insulin. When exposed to high concentrations of insulin (10 μM), co-cultured adipocytes showed decreased intracellular TG content after 9 days, suggesting increased lipolysis, a physiological trend observed in obesity and in T2DM. Adipocyte cultures alone and those exposed to high insulin concentrations however, did not show changes in TG content or lipolysis, suggesting that that endothelial cells may be important for producing more physiologically-relevant adipose models for studying obesity or T2DM.

In a similar model, Abbott et al. seeded liquified human lipoaspirates onto 3D silk scaffolds and determined adipocytes have increased glycerol and TG secretion compared to 2D culture [133]. In order to model the low-grade inflammation observed in obesity, the secretome of adipocytes exposed to TNFα in 2D and 3D culture was assessed (Fig. 2f) [133]. Interestingly, when treated with the pro-inflammatory cytokine, TNFα, adipocytes grown on 3D scaffolds secreted obesity-associated markers such as: IL1-α, osteoprotegerin (OPG), tissue inhibitor of metalloproteinases 2 (TIMP2), as well as T2DM related proteins such as IL-6, IL-8, and regulated-on-activation-normal-T-Cell-expressed-and-secreted (RANTES) [133]. In contrast to expectations and also the work by Aubin et al., TNF-α stimulation increased glucose uptake which the authors attribute to other cell types within the lipoaspirates, including endothelial cells [128, 150]. These data demonstrate that adipocytes grown on a silk scaffold and subject to acute inflammation, respond in a similar manner as to what is observed in obesity and T2DM but the interactions between adipocytes and other cell types in the microenvironment are responsible for the full phenotype seen in patients.

Silk fibroin has also been used in a hydrogel format to bioprint scaffolds to support the differentiation of chondrocytes, osteocytes, and adipose tissue [134]. Das et al. showed that silk fibroin hydrogels bioprinted and crosslinked with tyrosinase supported the expression of *PPARG, CEBPα,* and *LPL* in adipocytes derived from human nasal inferior turbinate tissue-derived mesenchymal stromal cells. Because Das et al. focused on chondrogenesis and osteogenesis, the morphology and function of adipocytes differentiated on this matrix are unclear. It would be interesting to see how their method directly compares to other solid silk scaffold and hydrogel-based models.

The above models primarily focused on adipocyte biology with and without endothelial cells. However, in 2016, Proulx et al. analyzed the influence of adipocytes exposed to TNF and IL-1β on endothelial cells derived from human microvascular endothelial cells using the model established by Aubin et al. [129, 135]. Intriguingly, capillary-like structures made of endothelial cells exposed to TNF-α or IL-1β were shorter, less branched and were more apoptotic; demonstrating a valid model to study the crosstalk between adipocytes and endothelial cells. Indeed, a follow-up study by Proulx et al. in 2018 implanted these models in nude mice for 14 days and found that the human endothelial cells integrated with the host vasculature within 3 days [136]. Proulx et al. and the aforementioned models provide crucial insights into the complexity associated with obesity and T2DM that 1 day may be translated into the clinic.

### Cancer

As researchers learn more about the contributions of obesity and adipose tissue to cancer progression, tissue-engineered adipose models are becoming increasingly useful and necessary to interrogate the interactions between cancer cells and adipose tissues. (For more on 3D tissue-engineered in vitro models of cancer in bone, please see previous reviews [5, 151].) In designing 3D adipose-cancer models, researchers must consider parameters such as: cell type/source, concentration, and ratios; scaffold material properties and pore size; media composition; imaging modalities desired; and potential bioreactor or other influences of stress or strain on the model. Interestingly, it appears that gravitational forces can also affect differentiation of BM-MSCs into osteogenic or adipogenic lineages, so this may be another parameter that could be altered, if necessary [152].

The most common 3D adipose/cancer models that have been developed are used to study breast cancer, although a few models have also been developed to study adipocyte-prostate cancer cell interactions and bone marrow adipocyte-myeloma cell interactions. These model systems will be described here.

For a review of synthetic adipose model systems for breast tissue engineering applications developed before 2010, please see the following review [153]. Since 2010, there have been substantial advances in model systems, and in 2014 a model was even developed to recapitulate mammary gland involution using engineered porous collagen/HA matrices [139]. This study used a mouse mammary epithelial cell line (KIM-2) and mouse 3 T3-L1 pre-adipocytes that were differentiated into adipocytes. This model could easily be adapted to study carcinogenesis.

One 3D model that has been used to study breast cancer is a microfluidic system with human breast cancer cells (MCF-7) and primary ASCs, although these were not differentiated into adipocytes (though the possibility exists). The model system was the first to demonstrate the potential use of a microfluidic-based in vitro 3D breast cancer model for effective evaluation of photodynamic therapy [137]. Their highly controllable model had the capability for real-time monitoring of tissue progression, implementing a circulation-like dynamic flow of media, drug testing and investigating the relationship between light penetration and tissue depth in photodynamic therapy [137]. This highly innovative model may be used to study adipocyte-breast cancer cell interactions in future work. The 3D nature of this model was a result of the chamber depth (200 μm), which allowed the stromal and cancer cells to produce their own ECM by self-organization, although the components of this ECM were not characterized in this study.

Another useful 3D model system is the human decellularized adipose tissue scaffold, which has been used to model breast cancer growth and drug response in vitro [138]. The benefit of these models is that they have similar ECM composition to the microenvironment of human breast tissue and recapitulate in vivo results much more closely than 2D models can, but they do not include live adipocytes currently [138]. An easy next step would be co-culturing adipocytes with breast cancer cells in these models. Similarly, silk scaffolds have been used to culture breast cancer cells in a 3D microenvironment [154]. Although no breast cancer and adipocyte co-cultures on silk have been described, many adipose tissue silk cultures have been described (see above) and thus silk scaffold adipose and silk scaffold breast cancer cell models could easily be combined to form breast cancer/adipocyte co-cultures on silk matrices.

In 2018, Hume et al. described a model using engineered collagen I scaffolds seeded with hMSCs that were differentiated into mature adipocytes [140]. The model supported co-culture with the breast cancer cell line MDA-MB-231, for 7 days and allowed for multiphoton microscopy analysis. Greater migration of cancer cells was observed in adipose-containing models versus empty, control scaffolds, demonstrating their utility in examining adipocyte contributions to tumor cell homing or metastasis. This model was then built upon to study tumor fragment cultures by the same group [141]. This model was used to distinguish tumor cell migration phenotypes and to test therapeutic agents for their effect on tumor cell migration. Clear, unobstructed brain/body imaging cocktails and computational analysis (CUBIC) optical clearing methods were utilized to make the samples optically transparent so that the responses of tumor cells could be more easily assessed. These exciting models may be used to explore even more about adipocyte-tumor cell crosstalk in future studies.

A new model using collagen-glycosaminoglycan-chitosan scaffolds and mature adipocytes differentiated from hASCs was developed in 2018 [142]. The investigators found that MCF-7 breast cancer cells were less sensitive to the anti-proliferative effects of tamoxifen when co-cultured with adipocytes derived from obese rather than normal-BMI women. These results suggested the tamoxifen resistance is in part due to differences in IL-6, leptin, and TNFα between adipose from leaner and obese women’s hASCs [142]. This interesting finding highlights the utility of new models to bring new insights into the ways in which adipocytes and tumor cells interact and the role of obesity in altering that relationship. This work was based on a previous publication from 2013 where the fatty-tissue equivalent was developed, breast cancer cells were found to change the process of adipogenesis, and many genes were differentially expressed by breast cancer cells when cultured with adipose models versus controls [155]. These publications have thus revealed the numerous ways in which adipocytes and tumor cells partake in crosstalk and bidirectional signaling.

**Fig. 3:**
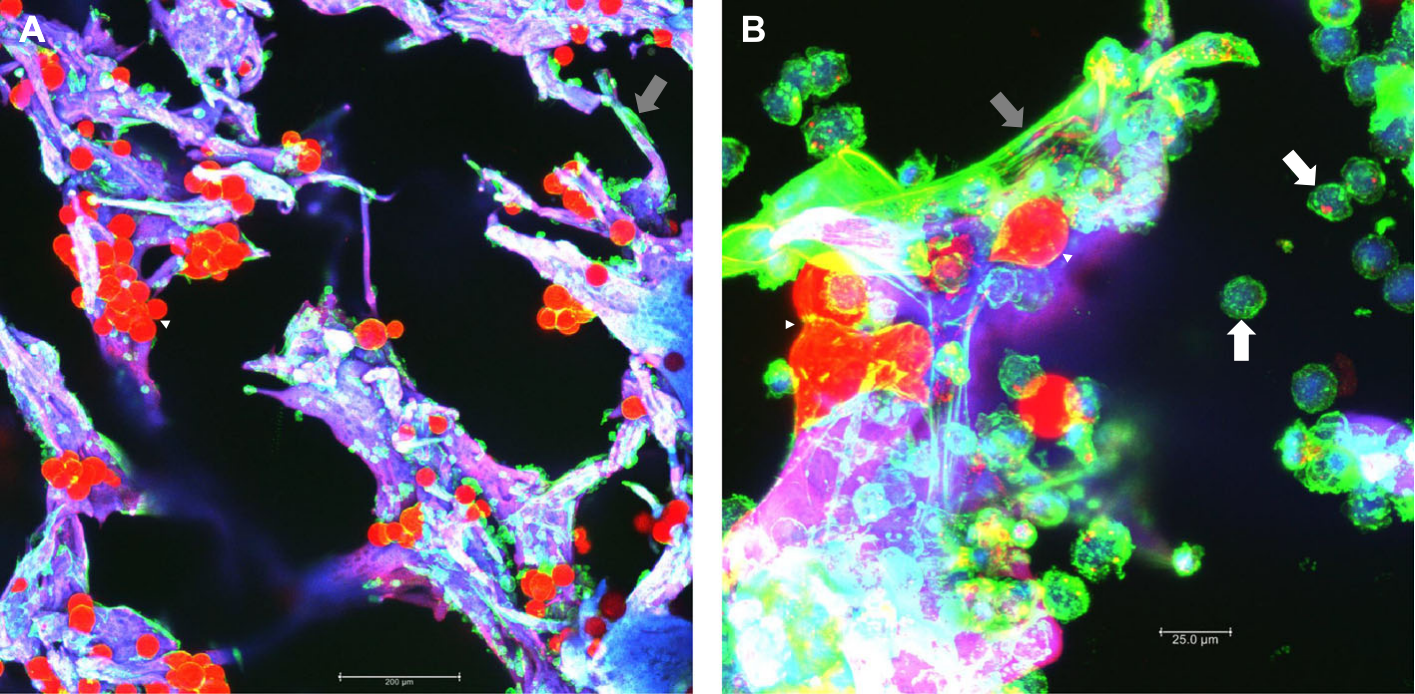
Example of tissue-engineered bone marrow adipose tissue. Silk scaffolds were used as a platform to make 3D, tissue-engineered bone marrow adipose tissue (BMAT). **a** Mouse BMAT was differentiated from male, 10-month-old KaLwRij mouse-derived bone marrow mesenchymal stem cells (BM-MSCs). Briefly, BM-MSCs were seeded onto scaffolds and cultured for 6 days, then put into adipogenic media for 10 days, put into a maintenance media for 1 week, and finally imaged. Scale bar = 200 μm. **b** Mouse-derived BM-MSCs were treated as above up until adipogenesis then the mouse myeloma cell line, 5TGM1s, were co-culture with the differentiated adipocytes for 1 week in maintenance media. Scale bar = 25.0 μm. Samples were stained Oil Red O (red) and phalloidin/actin (green). The scaffolds are autofluorescent in all channels and thus appear purple. Imaging was performed with a confocal microscope using maximum projections of Z-stacked images. Many adipocytes are visible (red, white arrowheads), along with many undifferentiated stromal cells (grey arrows). Rounded green myeloma cells are seen throughout the scaffold (white arrows)

In addition to breast cancer, prostate cancer-adipocyte 3D and myeloma cell-adipocyte models have also been developed. In 2018, Mosaad et al. published a high-throughput microtissue culture system where 3D cultures were formed using a novel microwell platform, the *Microwell-mesh* [143]*. Adipocytes were differentiated from hMSCs and added to f*irefly luciferase-expressing C42B prostate cancer cells *to create scaffold-free 3D cultures, or tumor cells were cultured above the adipocytes in a transwell and assessed for migration*. Interestingly, 3D adipocyte cultures decreased prostate cancer cell chemotaxis significantly, while 2D adipocyte cultures induced a slight increase in PC3 prostate cancer cell chemotaxis, highlighting the differences in secreted factors from 2D and 3D adipocytes [143]. Also, docetaxel drug resistance was seen in C42B prostate cancer cells when cultured in 2D with hMSCs or adipocytes, but drug resistance was not seen with adipocytes when cultured in 3D, as assessed by bioluminescence [143]. In another model of prostate cancer from the Podgorski laboratory, prostate tumor spheroids were grown on reconstituted basement membranes and cultured alone or in transwell with bone marrow adipocytes [144]. The adipocytes increased the prostate tumor spheroid after 3 and 5 days in co-culture. The group also included bone marrow macrophages into some of their 3D hydrogel cultures and used the models to study prostate cancer cell response to therapies and migration patterns [144]. Our laboratory has developed a 3D BMAT model that uses silk scaffolds, adipocytes derived from mouse or human BM-MSCs, and multiple myeloma (MM) cell lines (Fig. 3a and b) [4, 156]. By comparing 2D to 3D culture, we observed that 3D BMAT was much more physiologically relevant than 2D BMAT. Microarray analysis of MSCs differentiated for 28 days on 3D silk scaffolds revealed that pathways such as DNA replication, ribosome biosynthesis, cell cycle, and metabolic pathways were significantly enriched relative to 2D culture [4]. Conversely, pathways related to inflammation (NOD-like receptor), disease (malaria and prion-related diseases), and cytokines (TNF) were significantly enriched in 2D BMAT. Parallel proteomic analysis revealed similar patterns as the microarray data [4]. Given the intimate link between inflammation and obesity, T2DM and cancer, the perception of healthy control AT in traditional 2D cell culture may be closer phenotypically to the inflammatory disease phenotypes that are being studied. The use of our 3D silk model and other models like it will allow a more refined definition of “healthy” and disease-state adipose tissue. We also observed that adipocytes supported MM cell growth and that MM cells induced delipidation of BM adipocytes, through processes that are as of yet unknown but that we are investigating [4]. Overall, by using 3D models of prostate, breast, or myeloma cancer cells and adipocytes, researchers have elucidated new mechanisms of tumor-host interaction, which represent important and useful research directions to pursue.

## Conclusions

Herein, we reviewed the efforts of researchers across varying disciplines to recapitulate the complex picture of adipocytes in their natural environment using 3D bioengineered environments. Human and mouse precursors plumped up as they expressed PPAR-γ, adiponectin, FABP4, and leptin on a diverse array of materials ranging from collagen, silk, and even to magnetically levitated spheres. The addition of perfusion further improved upon the phenotype of adipocytes reflecting their in vivo counterparts. To a degree, the behavior of adipocytes on 3D silk scaffolds recapitulated the hyperinsulinism that is observed in obese patients. Additionally, hydrogel, silk-based and scaffold-free co-culture experiments with adipocytes highlighted the supportive role endothelial cells have in reflecting the in vivo environment and pave the way for modeling PVAT. With “omics” technology being more accessible, it has now been applied to the tissue engineering field to identify global expression and secretomes of adipose tissue on various types of scaffolds. These techniques have also been applied to co-culture experiments with adipocytes and various types of cancer to elucidate the bi-directional effects of each cell type. Modeling BAT on a photocrosslinkable hyaluronic acid-based scaffold proved to be a promising effort to explore the role of BAT in obesity and beyond. It may be interesting to use scaffolds in conjunction with a technique by the Brown lab, which used induced pluripotent stem cells to generate a renewable source of beige adipocytes [157]. One could imagine growing and implanting these cells to treat patients with metabolic disorders. The use of 3D models could also be applied to studying lipodystrophies associated with certain pharmaceuticals, genetic conditions, human immunodeficiency, viral infections, and beyond [158]. While great strides have been made in the tissue-engineered adipose field, exploration into immune cell recruitment to hypertrophic adipocytes, the status of hypoxia signaling within modeled tissue, and the effects of other engineered tissues like the pancreas, may create a more holistic disease model for obesity and T2DM.

The majority of 3D models that analyze interactions between cancer cells and adipocytes focus on breast cancer, prostate cancer, and myeloma. These models lay down a foundation for investigating other cancer-adipocyte interactions like melanoma, acute myeloid leukemia, lipomas, and ovarian cancer. Additionally, cancer-associated cachexia could be studied in vitro via co-culture experiments with adipocytes, cancer cells, muscle cells, and other cell types of the researcher’s choice. 3D scaffolds offer an alternative to time and resource intensive animal models and have the potential for high-throughput analyses. Based on the literature reviewed here, the horizon of tissue-engineered adipose models is bright and will grow to meet the ever-increasing demand to understand and treat diseases like obesity, T2DM, and cancer.

## Data Availability

Data sharing not applicable to this article as no datasets were generated or analyzed during the current study.

## Abbreviations

2D: Two-dimensional
3D: Three-dimensional
ACC: Acetyl-CoA carboxylase
ACS: Acyl-CoA synthetase
ADIPOQ: Adiponectin
ANGPT1: Angiopoietin-1
ApoE: Apolipoprotein E
ATMs: Adipose tissue macrophages
ATP: Adenosine triphosphate
BAT: Brown adipose tissue
BMAT: Bone marrow adipose tissue
BMI: Body mass index
BM-MSCs: Bone marrow-derived mesenchymal stem cells
CCL: C-C motif chemokine ligand
CCR: Chemokine receptor
CEBPB: CCAAT Enhancer Binding Protein Beta
CUBIC: Clear, unobstructed brain/body imaging cocktails and computational analysis
DHT: Dihydrotestosterone
Dio2: Type 2 deiodinase
EDC: 1-Ethyl-3-[3-dimethylaminopropyl] carbodiimide hydrochloride
ELP: Elastin-like polypeptide
ER: Endoplasmic Reticulum
FABP4: Fatty acid binding protein 4
FFAs: Free fatty acids
FGF2: Fibroblast growth factor 2
GAGs: Glycosaminoglycans
HA: Hyaluronic acid
hASCs: Human adipose-derived stem cells
hBM-MSCs: Human bone marrow-derived mesenchymal stem cells
HDLs: High-density lipoproteins
HGF: Hepatocyte growth factor
IKK-β: I kappa B kinase beta
IL-10: Interleukin 10
IL-1α: Interleukin 1α
IL-1β: Interleukin 1β
IL-6: Interleukin 6
IL-8: Interleukin 8
JNK1: C-jun-N-terminal kinase
LD: Lipid droplet
LDH: Lactate dehydrogenase
LPL: Lipoprotein lipase
MAPK: Mitogen activated protein kinase
MCP-1: Monocyte chemoattractant protein-1
NFκB: Nuclear factor kappa-light-chain-enhancer of activated B cells
NHS: N-hydroxysuccinimide
OPG: Osteoprotegerin
PAI-1: Plasminogen activator inhibitor-1
PDMS: Polydimethylsiloxane
PEG-4A: 4arm polyethylene glycol
PET-CT: Positron-emission tomography and computed tomography
PGC-1α: Peroxisome proliferator-activated receptor gamma coactivator 1-alpha
PI3K: Phosphatidylinositol 3-kinase
PKA: Protein kinase A
PLA: Poly-lactic acid
PEI: Polyethyleneimine
PPAR-γ: Peroxisome proliferator-activated receptor gamma
PRDM16: PR domain containing 16
PVAT: Perivascular adipose tissue
RANTES: Regulated-on-activation-normal-T-Cell-expressed-and-secreted
SAT: Subcutaneous adipose tissue
SVF: Stromal vascular fraction
T2DM: Type 2 diabetes mellitus
TGs: Triacylglycerols
TIMP2: Tissue inhibitor of metalloproteinases 2
TNF-α: Tumor necrosis factor alpha
UCP1: Uncoupling protein 1
VAT: Visceral adipose tissue
VEGF: Vascular endothelial growth factor
WAT: White adipose tissue
